# Supplementary material for: Early life factors and their relevance to intima-media thickness of the common carotid artery in early adulthood
Source: PLoS One. 2020 May 19;15(5):e0233227. doi: 10.1371/journal.pone.0233227 (PMC7237005; doi:10.1371/journal.pone.0233227)
Supplement: S1 Table — Values are presented as means (SD), medians (IQR) or frequencies (percentage). Full breastfeeding defined as breast milk including water given to the child. (DOCX) [file pone.0233227.s001.docx]

**S1 Table. Early life characteristics of participants lost to follow up**

| **Variables** |  | **Males** |  | **Females** |
| --- | --- | --- | --- | --- |
| **Early life factors** | **N** |  | **N** |  |
| Maternal age at child birth (yrs) | 157 | 29.4 (4.2) | 177 | 29.8 (4.0) |
| Paternal age at child birth (yrs) | 145 | 32.7 (5.1) | 159 | 33.0 (6.2) |
| Pregnancy duration (wks) | 157 | 40 (40, 41) | 177 | 40 (39, 41) |
| Gestational weight gain (kg) | 150 | 13.2 (4.4) | 171 | 12.9 (4.2) |
| Birthweight (g) | 157 | 3561 (476) | 177 | 3445 (417) |
| Full breastfeeding | 135 |  | 144 |  |
| Never (0-2 weeks) |  | 53 (39.3%) |  | 45 (31.3%) |
| Short duration (3-17 weeks) |  | 40 (29.6%) |  | 60 (41.7%) |
| Long duration (>17 weeks) |  | 42 (31.1%) |  | 39 (27.0%) |
| Birth year | 157 | 1987 (1982, 1990) | 177 | 1987 (1983, 1991) |

Values are presented as means (SD), medians (IQR) or frequencies (percentage).

Full breastfeeding defined as breast milk including water given to the child.
