# Supplementary material for: Early life factors and their relevance to intima-media thickness of the common carotid artery in early adulthood
Source: PLoS One. 2020 May 19;15(5):e0233227. doi: 10.1371/journal.pone.0233227 (PMC7237005; doi:10.1371/journal.pone.0233227)
Supplement: S2 Table — Average IMT: average of means of right and left side intima media thickness (IMT). T: tertile, n: sample size in tertile. Linear trends (P trend) were obtained in linear regression models with IMT as a continuous variable. 1Values are medians (25th, 75th percentiles) of early life factors. 2Values are adjusted least squares means (95% CIs) of IMT. Model A adjusted for adult age at IMT measurement and the physician taking the IMT measurement. 3Model B additionally adjusted for birth year (residuals of birth year were calculated on age at IMT measurement). (DOCX) [file pone.0233227.s002.docx]

**S2 Table. Association of maternal or paternal age at child birth and IMT in young adulthood**

|  | **Average IMT (mm)** | | | |  |
| --- | --- | --- | --- | --- | --- |
|  | **N** |  |  |  | **P trend** |
| **Females** | **142** | **T1 (n=44)** | **T2 (n=50)** | **T3 (n=48)** |  |
| **Maternal age at child birth (yrs)^1^** |  | 27 (25, 28) | 30 (29, 31) | 34 (33, 36) |  |
| Model A**^2^** |  | 0.54 (0.53, 0.56) | 0.55 (0.54, 0.57) | 0.57 (0.56, 0.58) | 0.001 |
| Model B**^3^** |  | 0.54 (0.53, 0.56) | 0.55 (0.54, 0.56) | 0.57 (0.55, 0.58) | 0.003 |
| **Males** | **120** | **T1 (n=38)** | **T2 (n=47)** | **T3 (n=35)** |  |
| **Maternal age at child birth (yrs)^1^** |  | 26.5 (24, 28) | 30 (29, 32) | 35 (34, 37) |  |
| Model A**^2^** |  | 0.57 (0.55, 0.59) | 0.56 (0.54, 0.58) | 0.58 (0.56, 0.60) | 0.972 |
| Model B**^3^** |  | 0.57 (0.56, 0.59) | 0.56 (0.55, 0.58) | 0.57 (0.55, 0.59) | 0.160 |
| **Females** | **137** | **T1 (n=41)** | **T2 (n=49)** | **T3 (n=47)** |  |
| **Paternal age at child birth (yrs)^1^** |  | 28 (26, 29) | 32 (32, 33) | 38 (36, 40) |  |
| Model A**^2^** |  | 0.55 (0.54, 0.57) | 0.55 (0.54, 0.56) | 0.56 (0.55, 0.58) | 0.2 |
| Model B**^3^** |  | 0.55 (0.54, 0.57) | 0.55 (0.54, 0.56) | 0.56 (0.55, 0.58) | 0.2 |
| **Males** | **119** | **T1 (n=38)** | **T2 (n=43)** | **T3 (n=38)** |  |
| **Paternal age at child birth (yrs)^1^** |  | 29 (28, 30) | 33 (31, 34) | 38.5 (36, 41) |  |
| Model A**^2^** |  | 0.57 (0.55, 0.59) | 0.56 (0.54, 0.58) | 0.58 (0.56, 0.6) | 0.7 |
| Model B**^3^** |  | 0.57 (0.55, 0.59) | 0.57 (0.55, 0.58) | 0.57 (0.55, 0.59) | 0.1 |

Average IMT: average of means of right and left side intima media thickness (IMT).

T: tertile, n: sample size in tertile.

Linear trends (P trend) were obtained in linear regression models with IMT as a continuous variable.

**^1^**Values are medians (25th, 75th percentiles) of early life factors.

**^2^**Values are adjusted least squares means (95% CIs) of IMT. Model A adjusted for adult age at IMT measurement and the physician taking the IMT measurement.

**^3^**Model B additionally adjusted for birth year (residuals of birth year were calculated on age at IMT measurement).
