# Supplementary material for: Early life factors and their relevance to intima-media thickness of the common carotid artery in early adulthood
Source: PLoS One. 2020 May 19;15(5):e0233227. doi: 10.1371/journal.pone.0233227 (PMC7237005; doi:10.1371/journal.pone.0233227)
Supplement: S4 Table — Average IMT: average of means of right and left side intima media thickness (IMT). T: tertile, n: sample size in tertile. Linear trends (P trend) were obtained in linear regression models with IMT as a continuous variable. 1Values are medians (25th, 75th percentiles) of maternal age at child birth. 2Values are adjusted least squares means (95% CIs) of IMT. Model A adjusted for adult age at IMT measurement and the physician taking the IMT measurement. 3Model B additionally adjusted for birth year (residuals of birth year were calculated on age at IMT measurement). (DOCX) [file pone.0233227.s004.docx]

**S4 Table. Association of maternal age at child birth and IMT in young adulthood among females with data on physical activity**

|  | **Average IMT (mm)** | | | |  |
| --- | --- | --- | --- | --- | --- |
|  | **N** |  |  |  | **P trend** |
| **Females** | **123** | **T1 (n=32)** | **T2 (n=46)** | **T3 (n=45)** |  |
| **Maternal age at child birth (yrs)^1^** |  | **27 (25, 28)** | **30 (29, 31)** | **34 (33, 36)** |  |
| Model A**^2^** |  | 0.54 (0.53, 0.56) | 0.56 (0.54, 0.57) | 0.57 (0.56, 0.58) | 0.0017 |
| Model B**^3^** |  | 0.55 (0.53, 0.56) | 0.56 (0.54, 0.57) | 0.57 (0.56, 0.58) | 0.0016 |
| Model B including sport |  | 0.54 (0.53, 0.56) | 0.56 (0.54, 0.57) | 0.57 (0.56, 0.58) | 0.0014 |
| **Females** | **68** | **T1 (n=26)** | **T2 (n=18)** | **T3 (n=24)** |  |
| **Maternal age at child birth (yrs)^1^** |  | **28 (27, 29)** | **31 (30, 32)** | **36 (34, 37)** |  |
| Model A^2^ |  | 0.55 (0.53, 0.57) | 0.56 (0.54, 0.58) | 0.58 (0.56, 0.60) | 0.0144 |
| Model B^3^ |  | 0.55 (0.53, 0.57) | 0.56 (0.54, 0.58) | 0.58 (0.56, 0.60) | 0.0092 |
| Model B including energy expenditure during sport |  | 0.55 (0.53, 0.57) | 0.56 (0.54, 0.58) | 0.58 (0.56, 0.60) | 0.0095 |

Average IMT: average of means of right and left side intima media thickness (IMT)

T: tertile, n: sample size in tertile.

Linear trends (P trend) were obtained in linear regression models with IMT as a continuous variable.

**^1^**Values are medians (25th, 75th percentiles) of maternal age at child birth.

**^2^**Values are adjusted least squares means (95% CIs) of IMT. Model A adjusted for adult age at IMT measurement and the physician taking the IMT measurement.

**^3^**Model B additionally adjusted for birth year (residuals of birth year were calculated on age at IMT measurement).
