# Supplementary material for: Early life factors and their relevance to intima-media thickness of the common carotid artery in early adulthood
Source: PLoS One. 2020 May 19;15(5):e0233227. doi: 10.1371/journal.pone.0233227 (PMC7237005; doi:10.1371/journal.pone.0233227)
Supplement: S5 Table — Average IMT: average of means of right and left side intima media thickness (IMT). T: tertile, n: sample size in tertile. Linear trends (P trend) were obtained in linear regression models with IMT as a continuous variable. 1p values less than 0.025 are considered significant according to Bonferroni adjustment. 2Values are medians (25th, 75th percentiles) of early life factors. 3Values are adjusted least squares means (95% CIs) of IMT. Model A adjusted for adult age at IMT measurement and the physician taking the IMT measurement. 4Model B additionally adjusted for birth year (residuals of birth year were calculated on age at IMT measurement). (DOCX) [file pone.0233227.s005.docx]

**S5 Table. Association of pregnancy duration or gestational weight gain with IMT in young adulthood**

|  | **Average IMT (mm)** | | | | |
| --- | --- | --- | --- | --- | --- |
| **Early life factors** | **N** |  |  |  | **P trend^1^** |
|  |  | **T1 (n=68)** | **T2 (n=119)** | **T3 (n=78)** |  |
| **Pregnancy duration (wks)^2^** | **265** | 39 (38, 39) | 40 (40, 40) | 41 (41, 41) |  |
| Model A**^3^** |  | 0.56 (0.55, 0.57) | 0.56 (0.55, 0.57) | 0.56 (0.55, 0.58) | 0.7 |
| Model B**^4^** |  | 0.56 (0.55, 0.57) | 0.56 (0.55, 0.57) | 0.56 (0.55, 0.57) | 0.9 |
|  |  | **T1 (n=91)** | **T2 (n=85)** | **T3 (n=82)** |  |
| **Gestational weight gain (kg)^2^** | **258** | 10 (8, 10) | 13 (12, 13) | 16 (15, 18) |  |
| Model A**^3^** |  | 0.56 (0.54, 0.57) | 0.56 (0.55, 0.57) | 0.56 (0.55, 0.58) | 0.4 |
| Model B**^4^** |  | 0.56 (0.55, 0.57) | 0.56 (0.55, 0.57) | 0.56 (0.55, 0.57) | 0.5 |

Average IMT: average of means of right and left side intima media thickness (IMT).

T: tertile, n: sample size in tertile.

Linear trends (P trend) were obtained in linear regression models with IMT as a continuous variable.

^1^p values less than 0.025 are considered significant according to Bonferroni adjustment.

**^2^**Values are medians (25th, 75th percentiles) of early life factors.

**^3^**Values are adjusted least squares means (95% CIs) of IMT. Model A adjusted for adult age at IMT measurement and the physician taking the IMT measurement.

**^4^**Model B additionally adjusted for birth year (residuals of birth year were calculated on age at IMT measurement).
