# Supplementary material for: Early life factors and their relevance to intima-media thickness of the common carotid artery in early adulthood
Source: PLoS One. 2020 May 19;15(5):e0233227. doi: 10.1371/journal.pone.0233227 (PMC7237005; doi:10.1371/journal.pone.0233227)
Supplement: S6 Table — AGA: appropriate for gestational age, LGA: large for gestational age, SGA: small for gestational age. AGA, LGA and SGA defined according to German sex-specific birth weight and length-for-gestational-age curves. Linear trends (P difference) were obtained in linear regression models with IMT as a continuous variable and birthweight according to gestational age as a 3 level categorical variable (0 = SGA; 1 = AGA; 2 = LGA with AGA set as the reference category in the models). 1p difference and trend less than 0.025 are considered significant according to Bonferroni adjustment. 2Values are frequencies (percentages) of birthweight according to gestational age. 3Values are adjusted least squares means (95% CIs) of IMT. 4Values are medians (25th, 75th percentiles) of birthweight. Model A adjusted for adult age at IMT measurement and the physician taking the IMT measurement. Model B additionally adjusted for birth year (residuals of birth year were calculated on age at IMT measurement). (DOCX) [file pone.0233227.s006.docx]

**S6 Table. Association of birthweight for gestational age or birthweight and adult IMT**

| **Early life factors** | **Average IMT (mm)** | | | |
| --- | --- | --- | --- | --- |
| **Birthweight according to gestational age** | **SGA** | **AGA** | **LGA** |  |
| **N (%)^2^** | **n=28 (10.6)** | **n=203 (76.6)** | **n=34 (12.8)** | **P difference**^1^ |
| Model A**^3^** | 0.55 (0.53, 0.57) | 0.56 (0.55, 0.57) | 0.56 (0.54, 0.58) | 0.8 |
| Model B | 0.55 (0.54, 0.57) | 0.56 (0.55, 0.57) | 0.56 (0.54, 0.58) | 0.8 |
|  | **T1 (n=89)** | **T2 (n=88)** | **T3 (n=88)** | **P trend**^1^ |
| **Birthweight (kg)^4^** | 3040 (2900, 3140) | 3455 (3385, 3550) | 3900 (3800, 4165) |  |
| Model A**^3^** | 0.56 (0.55, 0.57) | 0.56 (0.55, 0.57) | 0.56 (0.55, 0.57) | 0.7 |
| Model B | 0.56 (0.55, 0.57) | 0.56 (0.55, 0.57) | 0.56 (0.55, 0.57) | 0.9 |

AGA: appropriate for gestational age, LGA: large for gestational age, SGA: small for gestational age.

AGA, LGA and SGA defined according to German sex-specific birth weight and length-for-gestational-age curves.

Linear trends (P difference) were obtained in linear regression models with IMT as a continuous variable and birthweight according to gestational age as a 3 level categorical variable (0=SGA; 1=AGA; 2=LGA with AGA set as the reference category in the models).

^1^p difference and trend less than 0.025 are considered significant according to Bonferroni adjustment.

**^2^**Values are frequencies (percentages) of birthweight according to gestational age.

**^3^**Values are adjusted least squares means (95% CIs) of IMT.

**^4^**Values are medians (25th, 75th percentiles) of birthweight.

Model A adjusted for adult age at IMT measurement and the physician taking the IMT measurement.

Model B additionally adjusted for birth year (residuals of birth year were calculated on age at IMT measurement).
